# Poly[bis­[μ_2_-1,4-bis­(1,2,4-triazol-1-yl­meth­yl)benzene-κ^2^ *N* ^4^:*N* ^4′^]bis(nitrito-κ*O*)cobalt(II)]

**DOI:** 10.1107/S1600536809039415

**Published:** 2009-12-19

**Authors:** Xia Zhu, Ying Guo, Yun-Ling Zou

**Affiliations:** a Science College, Civil Aviation University of China, Tianjin 300300, People’s Republic of China

## Abstract

The Co^II^ atom in the title complex, [Co(NO_2_)_2_(C_12_H_12_N_6_)_2_]_*n*_, lies on an inversion center and is coordinated by four N atoms from the triazole rings of two symmetry-related pairs of 1,4-bis­(1,2,4-triazol-1-ylmeth­yl)benzene (bbtz) ligands and two O atoms from two symmetry-related monodentate nitrate ligands in a distorted octa­hedral geometry. The Co atoms are bridged by four bbtz ligands, forming a two-dimensional (4,4) network parallel to (102).

## Related literature

The synthesis of the ligand 1,4-bis­(1,2,4-triazol-1-ylmeth­yl)-benzene (bbtz) was described by Peng *et al.* (2004 ▶). Several bbtz complexes have been synthesized and structurally characterized, see: Li *et al.* (2005 ▶); Wang *et al.* (2007 ▶).
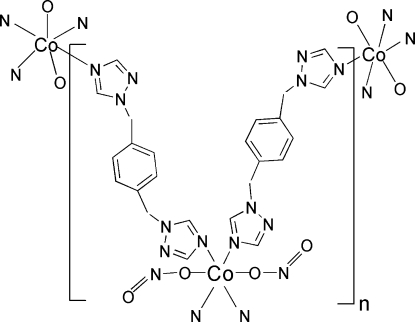


## Experimental

### 

#### Crystal data

[Co(NO_2_)_2_(C_12_H_12_N_6_)_2_]
*M* *_r_* = 631.50
Monoclinic, 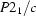

*a* = 8.3037 (13) Å
*b* = 20.376 (3) Å
*c* = 8.4261 (11) Å
β = 104.390 (4)°
*V* = 1380.9 (3) Å^3^
*Z* = 2
Mo *K*α radiation
μ = 0.68 mm^−1^
*T* = 193 K
0.33 × 0.26 × 0.10 mm

#### Data collection

Rigaku Mercury CCD diffractometer
Absorption correction: multi-scan (*REQAB*; Jacobson, 1998 ▶) *T*_min_ = 0.806, *T*_max_ = 0.935
15379 measured reflections
3152 independent reflections
2768 reflections with *I* > 2σ(*I*)
*R*_int_ = 0.032

#### Refinement

*R*[*F* ^2^ > 2σ(*F* ^2^)] = 0.041
*wR*(*F* ^2^) = 0.098
*S* = 1.07
3152 reflections
197 parameters
H-atom parameters constrained
Δρ_max_ = 0.28 e Å^−3^
Δρ_min_ = −0.29 e Å^−3^

### 

Data collection: *CrystalClear* (Rigaku, 2000 ▶); cell refinement: *CrystalClear*; data reduction: *CrystalClear*; program(s) used to solve structure: *SHELXS97* (Sheldrick, 2008 ▶); program(s) used to refine structure: *SHELXL97* (Sheldrick, 2008 ▶); molecular graphics: *SHELXTL* (Sheldrick, 2008 ▶); software used to prepare material for publication: *SHELXTL*.

## Supplementary Material

Crystal structure: contains datablocks I, global. DOI: 10.1107/S1600536809039415/gk2227sup1.cif

Structure factors: contains datablocks I. DOI: 10.1107/S1600536809039415/gk2227Isup2.hkl

Additional supplementary materials:  crystallographic information; 3D view; checkCIF report

## Figures and Tables

**Table:** 

Co1—O1	2.1031 (15)
Co1—N6^i^	2.1418 (16)
Co1—N3	2.1530 (16)

**Table:** 

O1^ii^—Co1—O1	180
O1—Co1—N6^i^	85.99 (6)
N6^i^—Co1—N6^iii^	180
O1—Co1—N3	93.70 (6)
N6^i^—Co1—N3	90.52 (6)
N3—Co1—N3^ii^	180

Symmetry codes: (i) 
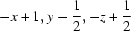
; (ii) 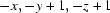; (iii) 
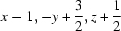
.

**Fig. 1.**
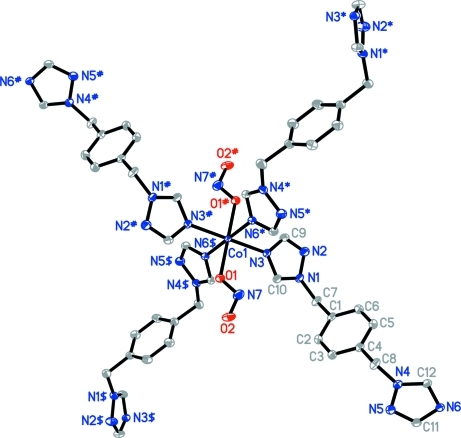
The coordination environment of the CoII atom in the title compound. Ellipsoids are drawn at the 30% probability level. [Symmetry codes # -x, -y + 1, -z + 1; $ -x + 1, y - 1/2, -z + 1/2; * x - 1, -y + 3/2, z + 1/2]. The hydrogen atoms have been omitted for clarity.

**Fig. 2.**
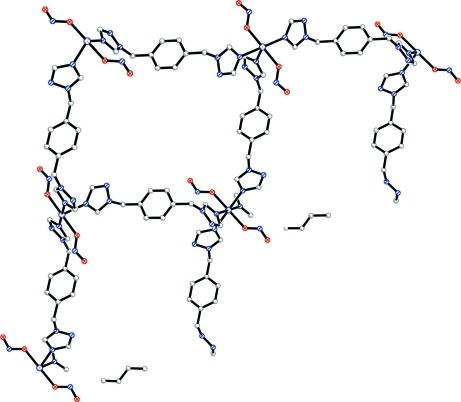
View of the two-dimensional (4,4) network of the title compound along the c direction.

**Fig. 3.**
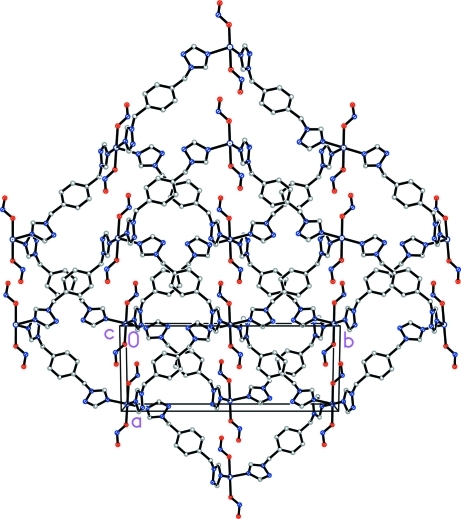
The cell packing of the title compound.

## supplementary crystallographic information

### Comment

The title compound is isostructural with its azido Ni^II ^analog (Wang *et al.*, 2007).

Fig. 1 shows the local coordination of the Co^II^ atom. In the complex the Co^II^ atom occupies an inversion center. The coordination geometry of the Co^II^ atom is a distorted octahedron. Each Co^II^ atom is coordinated equatorial by four nitrogen atoms from the triazole rings of four bbtz ligands [Co1—N3, 2.1530 (16) Å; Co1—N6 (-*x* + 1, *y* - 1/2, -*z* + 1/2), 2.1418 (16) Å], and axially by two oxygen atoms from two symmetry-related nitrite anions [Co1—O1, 2.1031 (15) Å]. The bbtz ligands shows the *trans-gauche* conformation, similar to the uncoordinated bbtz molecule (Peng *et al.*, 2004).

As illustrated in Fig.2, each bbtz ligand coordinated to the Co^II^ atoms through its two triazole nitrogen atoms, thus acting as a bridging bidentate ligand to form a two-dimensional (4,4) network. As a consequence of the symmetry of the crystal structure, the edge lengths are equal, with a vaule of 14.4182 (14) Å. The square-grid sheets are stacked in an offset fashion parallel to the c direction. The off-set half-cell superposition of each pair of adjacent networks divides the voids into smaller rectangle. The nitrile anions of one sheet project into the holes of the next sheet. In the superposition structure, the sheets are arranged in the sequence^···^A—B—A—B^···^ (Fig.3).

### Experimental

A H_2_O/MeOH solution (20 ml, 1:1 *v*/*v*) of Co(ClO_4_)_2_^.^6H_2_O (0.50 mmol) and NaNO_2_ (2.0 mmol) was added to one leg of a "H-shaped" tube, and a H_2_O/MeOH solution (20 ml, 1:1 *v*/*v*) of bbtz (0.240 g, 1.00 mmol) was added to the other leg of the tube. After several weeks, the well shaped red single crystals were obtained. Found: C, 45.57; H, 3.79; N, 30.94%. Calcd. for C_24_H_24Co_N_14_O_4_: C, 45.65; H, 3.83; N, 31.06%.

### Refinement

H atom were placed in idealized positions and refined as riding, with C—H distances of 0.95 (triazole and benzene) and 0.99Å (methyl), and with *U*_iso_(H) = 1.2 times *U*_eq_(C).

### Crystal data

**Table:**

[Co(NO_2_)_2_(C_12_H_12_N_6_)_2_]	*F*(000) = 650
*M**_r_* = 631.50	*D*_x_ = 1.519 Mg m^−^^3^
Monoclinic, *P*2_1_/*c*	Mo *K*α radiation, λ = 0.71073 Å
*a* = 8.3037 (13) Å	Cell parameters from 5616 reflections
*b* = 20.376 (3) Å	θ = 3.1–27.5°
*c* = 8.4261 (11) Å	µ = 0.68 mm^−^^1^
β = 104.390 (4)°	*T* = 193 K
*V* = 1380.9 (3) Å^3^	Block, red
*Z* = 2	0.33 × 0.26 × 0.10 mm

### Data collection

**Table:**

Rigaku Mercury CCD diffractometer	3152 independent reflections
Radiation source: fine-focus sealed tube	2768 reflections with *I* > 2σ(*I*)
graphite	*R*_int_ = 0.032
ω scans	θ_max_ = 27.5°, θ_min_ = 3.2°
Absorption correction: multi-scan (REQAB; Jacobson, 1998)	*h* = −10→10
*T*_min_ = 0.806, *T*_max_ = 0.935	*k* = −26→26
15379 measured reflections	*l* = −10→10

### Refinement

**Table:**

Refinement on *F*^2^	Primary atom site location: structure-invariant direct methods
Least-squares matrix: full	Secondary atom site location: difference Fourier map
*R*[*F*^2^ > 2σ(*F*^2^)] = 0.041	Hydrogen site location: inferred from neighbouring sites
*wR*(*F*^2^) = 0.098	H-atom parameters constrained
*S* = 1.07	*w* = 1/[σ^2^(*F*_o_^2^) + (0.0436*P*)^2^ + 0.7485*P*] where *P* = (*F*_o_^2^ + 2*F*_c_^2^)/3
3152 reflections	(Δ/σ)_max_ < 0.001
197 parameters	Δρ_max_ = 0.28 e Å^−^^3^
0 restraints	Δρ_min_ = −0.29 e Å^−^^3^

### Special details

**Table:**

Geometry. All e.s.d.'s (except the e.s.d. in the dihedral angle between two l.s. planes) are estimated using the full covariance matrix. The cell e.s.d.'s are taken into account individually in the estimation of e.s.d.'s in distances, angles and torsion angles; correlations between e.s.d.'s in cell parameters are only used when they are defined by crystal symmetry. An approximate (isotropic) treatment of cell e.s.d.'s is used for estimating e.s.d.'s involving l.s. planes.
Refinement. Refinement of *F*^2^ against ALL reflections. The weighted *R*-factor *wR* and goodness of fit *S* are based on *F*^2^, conventional *R*-factors *R* are based on *F*, with *F* set to zero for negative *F*^2^. The threshold expression of *F*^2^ > σ(*F*^2^) is used only for calculating *R*-factors(gt) *etc*. and is not relevant to the choice of reflections for refinement. *R*-factors based on *F*^2^ are statistically about twice as large as those based on *F*, and *R*- factors based on ALL data will be even larger.

### Fractional atomic coordinates and isotropic or equivalent isotropic displacement parameters (Å^2^)

**Table:**

	*x*	*y*	*z*	*U*_iso_*/*U*_eq_	
Co1	0.0000	0.5000	0.5000	0.02085 (12)	
O1	0.25015 (18)	0.50405 (7)	0.6333 (2)	0.0350 (4)	
O2	0.48900 (19)	0.54314 (9)	0.7278 (2)	0.0461 (4)	
N1	0.1620 (2)	0.57944 (8)	0.0939 (2)	0.0273 (4)	
N2	−0.0036 (2)	0.58502 (11)	0.0290 (2)	0.0412 (5)	
N3	0.0484 (2)	0.54604 (8)	0.2865 (2)	0.0257 (3)	
N4	0.8222 (2)	0.83452 (8)	0.2102 (2)	0.0274 (4)	
N5	0.9542 (2)	0.80233 (9)	0.1784 (2)	0.0349 (4)	
N6	0.93982 (19)	0.90596 (8)	0.08323 (19)	0.0253 (3)	
N7	0.3509 (2)	0.54893 (10)	0.6299 (2)	0.0425 (5)	
C1	0.3965 (2)	0.65065 (10)	0.0737 (2)	0.0282 (4)	
C2	0.5418 (3)	0.63661 (11)	0.1894 (3)	0.0343 (5)	
H2A	0.5699	0.5923	0.2188	0.041*	
C3	0.6470 (3)	0.68667 (11)	0.2632 (3)	0.0339 (5)	
H3A	0.7471	0.6762	0.3419	0.041*	
C4	0.6084 (3)	0.75136 (10)	0.2239 (2)	0.0289 (4)	
C5	0.4628 (3)	0.76574 (11)	0.1061 (3)	0.0376 (5)	
H5A	0.4353	0.8101	0.0766	0.045*	
C6	0.3576 (3)	0.71570 (11)	0.0315 (3)	0.0364 (5)	
H6A	0.2585	0.7260	−0.0488	0.044*	
C7	0.2823 (3)	0.59545 (11)	−0.0029 (3)	0.0333 (5)	
H7A	0.2214	0.6082	−0.1151	0.040*	
H7B	0.3494	0.5560	−0.0112	0.040*	
C8	0.7201 (3)	0.80549 (11)	0.3108 (3)	0.0370 (5)	
H8A	0.6508	0.8403	0.3425	0.044*	
H8B	0.7939	0.7877	0.4125	0.044*	
C9	−0.0655 (3)	0.56403 (12)	0.1496 (3)	0.0371 (5)	
H9A	−0.1817	0.5618	0.1406	0.044*	
C10	0.1907 (3)	0.55662 (11)	0.2456 (2)	0.0312 (5)	
H10A	0.2979	0.5489	0.3153	0.037*	
C11	1.0198 (3)	0.84752 (10)	0.1016 (3)	0.0317 (5)	
H11A	1.1157	0.8397	0.0621	0.038*	
C12	0.8157 (2)	0.89541 (10)	0.1543 (2)	0.0269 (4)	
H12A	0.7344	0.9270	0.1635	0.032*	

### Atomic displacement parameters (Å^2^)

**Table:**

	*U*^11^	*U*^22^	*U*^33^	*U*^12^	*U*^13^	*U*^23^
Co1	0.01814 (19)	0.02220 (19)	0.0236 (2)	−0.00012 (13)	0.00787 (14)	0.00201 (14)
O1	0.0210 (7)	0.0348 (8)	0.0459 (9)	−0.0049 (6)	0.0020 (6)	0.0075 (7)
O2	0.0229 (8)	0.0624 (11)	0.0527 (10)	−0.0088 (7)	0.0087 (7)	−0.0060 (9)
N1	0.0262 (9)	0.0307 (9)	0.0257 (8)	−0.0063 (7)	0.0079 (7)	0.0017 (7)
N2	0.0296 (10)	0.0549 (13)	0.0378 (10)	−0.0010 (9)	0.0058 (8)	0.0142 (9)
N3	0.0251 (8)	0.0266 (8)	0.0273 (8)	0.0002 (6)	0.0101 (6)	0.0036 (7)
N4	0.0262 (8)	0.0276 (8)	0.0291 (9)	−0.0053 (7)	0.0083 (7)	−0.0024 (7)
N5	0.0296 (9)	0.0304 (9)	0.0455 (11)	0.0004 (7)	0.0108 (8)	0.0020 (8)
N6	0.0221 (8)	0.0260 (8)	0.0289 (8)	−0.0026 (6)	0.0084 (6)	−0.0019 (7)
N7	0.0331 (10)	0.0523 (12)	0.0406 (11)	−0.0124 (9)	0.0061 (8)	0.0041 (9)
C1	0.0274 (10)	0.0323 (11)	0.0283 (10)	−0.0054 (8)	0.0135 (8)	0.0017 (8)
C2	0.0330 (11)	0.0293 (11)	0.0412 (12)	−0.0004 (9)	0.0101 (9)	0.0087 (9)
C3	0.0288 (11)	0.0365 (11)	0.0342 (11)	−0.0014 (9)	0.0034 (9)	0.0089 (9)
C4	0.0293 (10)	0.0314 (10)	0.0284 (10)	−0.0053 (8)	0.0118 (8)	0.0013 (8)
C5	0.0344 (12)	0.0282 (11)	0.0499 (14)	−0.0001 (9)	0.0098 (10)	0.0077 (10)
C6	0.0284 (11)	0.0389 (12)	0.0394 (12)	0.0001 (9)	0.0035 (9)	0.0091 (10)
C7	0.0368 (12)	0.0396 (12)	0.0281 (10)	−0.0116 (9)	0.0167 (9)	−0.0018 (9)
C8	0.0424 (13)	0.0397 (12)	0.0323 (11)	−0.0137 (10)	0.0154 (10)	−0.0017 (10)
C9	0.0244 (11)	0.0480 (13)	0.0404 (12)	0.0020 (9)	0.0110 (9)	0.0111 (10)
C10	0.0249 (10)	0.0411 (12)	0.0280 (10)	−0.0036 (8)	0.0069 (8)	0.0064 (9)
C11	0.0244 (10)	0.0303 (10)	0.0414 (12)	−0.0003 (8)	0.0099 (9)	−0.0019 (9)
C12	0.0242 (10)	0.0267 (10)	0.0309 (10)	−0.0022 (8)	0.0088 (8)	−0.0022 (8)

### Geometric parameters (Å, °)

**Table:**

Co1—O1^i^	2.1031 (15)	C1—C2	1.380 (3)
Co1—O1	2.1031 (15)	C1—C6	1.389 (3)
Co1—N6^ii^	2.1418 (16)	C1—C7	1.509 (3)
Co1—N6^iii^	2.1418 (16)	C2—C3	1.385 (3)
Co1—N3	2.1530 (16)	C2—H2A	0.9500
Co1—N3^i^	2.1530 (16)	C3—C4	1.377 (3)
O1—N7	1.245 (2)	C3—H3A	0.9500
O2—N7	1.240 (2)	C4—C5	1.391 (3)
N1—C10	1.325 (3)	C4—C8	1.509 (3)
N1—N2	1.353 (2)	C5—C6	1.387 (3)
N1—C7	1.475 (2)	C5—H5A	0.9500
N2—C9	1.319 (3)	C6—H6A	0.9500
N3—C10	1.328 (2)	C7—H7A	0.9900
N3—C9	1.348 (3)	C7—H7B	0.9900
N4—C12	1.324 (3)	C8—H8A	0.9900
N4—N5	1.360 (2)	C8—H8B	0.9900
N4—C8	1.465 (3)	C9—H9A	0.9500
N5—C11	1.319 (3)	C10—H10A	0.9500
N6—C12	1.331 (2)	C11—H11A	0.9500
N6—C11	1.353 (3)	C12—H12A	0.9500
N6—Co1^iv^	2.1418 (16)		
			
O1^i^—Co1—O1	180.0	C4—C3—C2	120.9 (2)
O1^i^—Co1—N6^ii^	94.01 (6)	C4—C3—H3A	119.6
O1—Co1—N6^ii^	85.99 (6)	C2—C3—H3A	119.6
O1^i^—Co1—N6^iii^	85.99 (6)	C3—C4—C5	118.83 (19)
O1—Co1—N6^iii^	94.01 (6)	C3—C4—C8	120.3 (2)
N6^ii^—Co1—N6^iii^	180.00 (8)	C5—C4—C8	120.85 (19)
O1^i^—Co1—N3	86.30 (6)	C6—C5—C4	120.4 (2)
O1—Co1—N3	93.70 (6)	C6—C5—H5A	119.8
N6^ii^—Co1—N3	90.52 (6)	C4—C5—H5A	119.8
N6^iii^—Co1—N3	89.48 (6)	C5—C6—C1	120.3 (2)
O1^i^—Co1—N3^i^	93.70 (6)	C5—C6—H6A	119.8
O1—Co1—N3^i^	86.30 (6)	C1—C6—H6A	119.8
N6^ii^—Co1—N3^i^	89.48 (6)	N1—C7—C1	111.64 (17)
N6^iii^—Co1—N3^i^	90.52 (6)	N1—C7—H7A	109.3
N3—Co1—N3^i^	180.00 (8)	C1—C7—H7A	109.3
N7—O1—Co1	126.67 (14)	N1—C7—H7B	109.3
C10—N1—N2	109.92 (16)	C1—C7—H7B	109.3
C10—N1—C7	128.82 (18)	H7A—C7—H7B	108.0
N2—N1—C7	121.23 (17)	N4—C8—C4	112.87 (17)
C9—N2—N1	102.28 (17)	N4—C8—H8A	109.0
C10—N3—C9	102.35 (17)	C4—C8—H8A	109.0
C10—N3—Co1	130.55 (14)	N4—C8—H8B	109.0
C9—N3—Co1	126.63 (14)	C4—C8—H8B	109.0
C12—N4—N5	110.21 (16)	H8A—C8—H8B	107.8
C12—N4—C8	127.34 (18)	N2—C9—N3	115.02 (19)
N5—N4—C8	122.01 (17)	N2—C9—H9A	122.5
C11—N5—N4	102.19 (17)	N3—C9—H9A	122.5
C12—N6—C11	102.66 (16)	N3—C10—N1	110.43 (18)
C12—N6—Co1^iv^	124.14 (13)	N3—C10—H10A	124.8
C11—N6—Co1^iv^	132.60 (13)	N1—C10—H10A	124.8
O2—N7—O1	115.52 (19)	N5—C11—N6	114.82 (18)
C2—C1—C6	119.04 (19)	N5—C11—H11A	122.6
C2—C1—C7	119.60 (19)	N6—C11—H11A	122.6
C6—C1—C7	121.36 (19)	N4—C12—N6	110.11 (17)
C1—C2—C3	120.5 (2)	N4—C12—H12A	124.9
C1—C2—H2A	119.7	N6—C12—H12A	124.9
C3—C2—H2A	119.7		
			
N6^ii^—Co1—O1—N7	126.75 (19)	C2—C1—C6—C5	−0.6 (3)
N6^iii^—Co1—O1—N7	−53.25 (19)	C7—C1—C6—C5	178.6 (2)
N3—Co1—O1—N7	36.48 (19)	C10—N1—C7—C1	−63.3 (3)
N3^i^—Co1—O1—N7	−143.52 (19)	N2—N1—C7—C1	119.1 (2)
C10—N1—N2—C9	−0.5 (2)	C2—C1—C7—N1	88.2 (2)
C7—N1—N2—C9	177.54 (19)	C6—C1—C7—N1	−91.0 (2)
O1^i^—Co1—N3—C10	−165.51 (19)	C12—N4—C8—C4	115.1 (2)
O1—Co1—N3—C10	14.49 (19)	N5—N4—C8—C4	−73.2 (3)
N6^ii^—Co1—N3—C10	−71.53 (19)	C3—C4—C8—N4	104.3 (2)
N6^iii^—Co1—N3—C10	108.47 (19)	C5—C4—C8—N4	−77.4 (3)
O1^i^—Co1—N3—C9	5.24 (18)	N1—N2—C9—N3	0.5 (3)
O1—Co1—N3—C9	−174.76 (18)	C10—N3—C9—N2	−0.3 (3)
N6^ii^—Co1—N3—C9	99.22 (18)	Co1—N3—C9—N2	−173.14 (16)
N6^iii^—Co1—N3—C9	−80.78 (18)	C9—N3—C10—N1	0.0 (2)
C12—N4—N5—C11	−0.5 (2)	Co1—N3—C10—N1	172.41 (13)
C8—N4—N5—C11	−173.46 (18)	N2—N1—C10—N3	0.3 (3)
Co1—O1—N7—O2	175.00 (14)	C7—N1—C10—N3	−177.51 (18)
C6—C1—C2—C3	0.3 (3)	N4—N5—C11—N6	0.4 (2)
C7—C1—C2—C3	−178.94 (19)	C12—N6—C11—N5	−0.1 (2)
C1—C2—C3—C4	0.6 (3)	Co1^iv^—N6—C11—N5	170.93 (14)
C2—C3—C4—C5	−1.2 (3)	N5—N4—C12—N6	0.5 (2)
C2—C3—C4—C8	177.2 (2)	C8—N4—C12—N6	172.95 (18)
C3—C4—C5—C6	0.9 (3)	C11—N6—C12—N4	−0.2 (2)
C8—C4—C5—C6	−177.4 (2)	Co1^iv^—N6—C12—N4	−172.27 (12)
C4—C5—C6—C1	0.0 (3)		

Symmetry codes: (i) −*x*, −*y*+1, −*z*+1; (ii) −*x*+1, *y*−1/2, −*z*+1/2; (iii) *x*−1, −*y*+3/2, *z*+1/2; (iv) −*x*+1, *y*+1/2, −*z*+1/2.

## References

[bb1] Jacobson, R. (1998). *REQAB* Private communication to Rigaku Corporation, Tokyo, Japan.

[bb2] Li, B. L., Peng, Y. F., Li, B. Z. & Zhang, Y. (2005). *Chem. Commun.* pp. 2333–2335. 10.1039/b418392d 15877119

[bb3] Peng, Y. F., Li, B. Z., Zzhou, J. H., Li, B. L. & Zhang, Y. (2004). *Chin. J. Struct. Chem.* **23**, 985–988.

[bb4] Rigaku (2000). *CrystalClear* Rigaku Corporation, Tokyo, Japan.

[bb5] Sheldrick, G. M. (2008). *Acta Cryst.* A**64**, 112–122. 10.1107/S0108767307043930 18156677

[bb6] Wang, L.-Y., Peng, Y.-F., Zhang, Y.-P., Li, B.-L. & Zhang, Y. (2007). *Acta Cryst.* C**63**, m297–m299. 10.1107/S0108270107021932 17609549

